# Clinical effectiveness of collaborative care for depression in UK primary care (CADET): cluster randomised controlled trial

**DOI:** 10.1136/bmj.f4913

**Published:** 2013-08-19

**Authors:** David A Richards, Jacqueline J Hill, Linda Gask, Karina Lovell, Carolyn Chew-Graham, Peter Bower, John Cape, Stephen Pilling, Ricardo Araya, David Kessler, J Martin Bland, Colin Green, Simon Gilbody, Glyn Lewis, Chris Manning, Adwoa Hughes-Morley, Michael Barkham

**Affiliations:** 1 Institute of Health Research, University of Exeter Medical School, Exeter EX1 2LU, UK; 2 School of Psychology, University of Exeter, Exeter, UK; 3 Centre for Primary Care, Institute of Population Health, Manchester Academic Health Science Centre, Williamson building, University of Manchester, Manchester, UK; 4 University of Manchester, School of Nursing, Midwifery & Social Work, Jean McFarlane Building, Manchester, UK; 5 Arthritis Research UK Primary Care Centre, Primary Care Sciences, Keele University, Keele, UK; 6 NIHR School for Primary Care Research, Centre for Primary Care, Institute of Population Health, Manchester Academic Health Science Centre, University of Manchester, UK; 7 Research Department of Clinical, Educational and Health Psychology, University College London, London, UK; 8 School of Social and Community Medicine, University of Bristol, Bristol, UK; 9 Department of Health Sciences, University of York, York, UK; 10 Institute of Health Research, University of Exeter Medical School, University of Exeter, UK; 11 Mental Health Research Group, Department of Health Sciences and Hull York Medical School, University of York, UK; 12 Mental Health Sciences Unit, University College London, UK; 13 Upstream Healthcare, Teddington, UK; 14 Centre for Psychological Services Research, Department of Psychology, University of Sheffield, Sheffield, UK

## Abstract

**Objective** To compare the clinical effectiveness of collaborative care with usual care in the management of patients with moderate to severe depression.

**Design** Cluster randomised controlled trial.

**Setting** 51 primary care practices in three primary care districts in the United Kingdom.

**Participants** 581 adults aged 18 years and older who met ICD-10 (international classification of diseases, 10th revision) criteria for a depressive episode on the revised Clinical Interview Schedule. We excluded acutely suicidal patients and those with psychosis, or with type I or type II bipolar disorder; patients whose low mood was associated with bereavement or whose primary presenting problem was alcohol or drug abuse; and patients receiving psychological treatment for their depression by specialist mental health services. We identified potentially eligible participants by searching computerised case records in general practices for patients with depression.

**Interventions** Collaborative care, including depression education, drug management, behavioural activation, relapse prevention, and primary care liaison, was delivered by care managers. Collaborative care involved six to 12 contacts with participants over 14 weeks, supervised by mental health specialists. Usual care was family doctors’ standard clinical practice.

**Main outcome measures** Depression symptoms (patient health questionnaire 9; PHQ-9), anxiety (generalised anxiety disorder 7; GAD-7), and quality of life (short form 36 questionnaire; SF-36) at four and 12 months; satisfaction with service quality (client satisfaction questionnaire; CSQ-8) at four months.

**Results** 276 participants were allocated to collaborative care and 305 allocated to usual care. At four months, mean depression score was 11.1 (standard deviation 7.3) for the collaborative care group and 12.7 (6.8) for the usual care group. After adjustment for baseline depression, mean depression score was 1.33 PHQ-9 points lower (95% confidence interval 0.35 to 2.31, P=0.009) in participants receiving collaborative care than in those receiving usual care at four months, and 1.36 points lower (0.07 to 2.64, P=0.04) at 12 months. Quality of mental health but not physical health was significantly better for collaborative care than for usual care at four months, but not 12 months. Anxiety did not differ between groups. Participants receiving collaborative care were significantly more satisfied with treatment than those receiving usual care. The number needed to treat for one patient to drop below the accepted diagnostic threshold for depression on the PHQ-9 was 8.4 immediately after treatment, and 6.5 at 12 months.

**Conclusions** Collaborative care has persistent positive effects up to 12 months after initiation of the intervention and is preferred by patients over usual care.

**Trial registration number** ISRCTN32829227.

## Introduction

Depression is a long term and relapsing condition, and is set to become the second largest cause of global disability by 2020.^1^ The responsibility for treatment in 90-95% of cases rests with primary care,^2^ but the organisation of care in this setting is not optimal for managing depression, with barriers between generalist and specialist professionals in mental health, poor patient adherence to pharmacological treatment,^3^ and limited specialist support for patients.^4^ Evidence is developing on the role of organisational interventions in improving the management of a range of chronic conditions. Their application to the management of depression includes “collaborative care,” a complex intervention developed in the United States incorporating a multiprofessional approach to patient care; a structured management plan; scheduled patient follow-ups; and enhanced interprofessional communication.^5^ In practice, this approach is achieved by the introduction of a care manager into primary care, responsible for delivering care to patients with depression under the supervision of a specialist, and for liaising between primary care doctors and mental health specialists.

Systematic reviews have shown that collaborative care improves depression outcomes, with some studies showing benefit for up to five years.^6^ ^7^ Previously, our 2006 systematic review of 28 collaborative care studies showed it to be effective (standardised mean difference −0.24, 95% confidence interval −0.17 to −0.32).^7^

However, most studies have been conducted in the US. In other areas of mental healthcare, organisational interventions developed in the US have not generalised outside the original healthcare context.^8^ For collaborative care, there is some supportive evidence from other contexts, including the developing world,^9^ ^10^ but uncertainty around the standardised effect size in trials in the United Kingdom (standardised mean difference 0.24, 95% confidence interval −0.060 to 0.547) and elsewhere.^7^ Owing to these limited non-US data and the relatively small effect size in trials of patients with depression alone, the UK National Institute for Health and Care Excellence (formerly the National Institute for Health and Clinical Excellence)^11^ issued a research recommendation for a fully powered UK evaluation of collaborative care.

There is considerable variation between study heterogeneity in terms of the duration and intensity of collaborative care, and in the training and background of care managers used in the reported studies. Therefore, we carefully developed our collaborative care intervention to apply outside the US, in healthcare systems with a well developed primary care sector.^12^ ^13^ ^14^ In our development work,^12^ we designed a care management intervention after systematic review,^7^ ^15^ in-depth qualitative interviews with patients, general practitioners, and mental health workers, and through consultations with the intervention originators in the US. In our phase II testing of this intervention,^13^ we found preliminary evidence indicating that collaborative care adapted to the UK was acceptable to patients and doctors, and could be effective outside the US, but that a cluster randomised controlled trial was needed to guard against potential contamination between trial arms.^13^ Consequently, we undertook a pragmatic cluster randomised trial to determine whether collaborative care is more clinically effective than usual care in the management of patients with moderate to severe depression.

## Methods

### Study design

The Clinical and Cost Effectiveness of Collaborative Care for Depression in UK Primary Care Trial (CADET) was a multicentre, two group, cluster randomised controlled trial.

### Setting and participants

We recruited participants between June 2009 and January 2011 from the electronic case records of primary care general practices in three UK sites: Bristol, London, and greater Manchester. Eligible participants were adults aged 18 years and older who met ICD-10 (international classification of diseases, 10th revision) criteria for a depressive episode when interviewed by research personnel using the revised Clinical Interview Schedule.^16^ We excluded acutely suicidal patients; those with psychosis, type I or type II bipolar disorder; those whose low mood was associated with bereavement or whose primary presenting problem was alcohol or drug abuse; and those receiving psychological treatment for their depression by specialist mental health services.

### Randomisation, concealment of allocation, and blinding

We randomly allocated primary care practices as they were recruited into the trial, minimised within sites by Index of Multiple Deprivation rank,^17^ number of general practitioners, and practice size. The allocation sequence was concealed from researchers recruiting practices and administered centrally using Minim.^18^ The Peninsula Clinical Trials Unit remotely managed participant identification and data collection. Research workers blind to allocation, assessed for eligibility and collected outcome measures using patients’ self report questionnaires to minimise the effect of potential unblinding. Owing to the nature of the intervention, it was not possible to blind participants, care managers, or general practitioners to allocations.

### Recruitment

We searched computerised case records from general practices for patients with at least one identification code in their electronic records. Several of such codes are widely used by general practitioners to classify patients as depressed. Practices contacted identified patients by letter or telephone to seek permission for researchers to contact them. Research staff interviewed potential participants that responded, to take consent and confirm eligibility via the revised Clinical Interview Schedule.

### Intervention and comparator groups

#### Intervention: collaborative care

Collaborative care, developed in our previous studies, was delivered by a team of care managers, supervised by mental health specialists. In addition to usual care from their general practitioners, care managers were to have six to 12 contacts with participants over 14 weeks: 30-40 minutes for an initial appointment face to face, followed by telephone contacts of 15-20 minutes thereafter.

Collaborative care consisted of antidepressant drug management, behavioural activation,^19^ symptom assessment, and communication between primary care physicians and the care managers. Care managers advised participants on their drug adherence and alerted primary care physicians to participant adherence or tolerance problems so that dose or agent could be amended. Behavioural activation^19^ is an effective form of brief cognitive behavioural therapy, which aims to disrupt depressive avoidance cycles and increase participants’ activity to provide more opportunity for exposure to positive mood reinforcing situations. Care managers assessed symptoms at each contact using the Hospital Anxiety and Depression Scale^20^ and discussed these with participants. Care managers also provided participants with relapse prevention advice.^21^ Finally, care managers provided general practitioners with regular updates and participant management advice at least monthly and more often if clinically indicated.

We recruited existing mental health workers in primary care with minimal or paraprofessional education as care managers, treating CADET participants alongside those patients being seen as part of their usual NHS role. We provided care managers with an additional five days’ training in collaborative care and weekly supervision by specialist professionals in mental health, including clinical psychologists, psychiatrists, academic general practitioners with special interest in mental health, or a senior nurse psychotherapist. Individual participants were discussed in supervision at least monthly, facilitated through a bespoke computerised system for patient management (www.pc-mis.co.uk), which automatically alerted supervisors and care managers of the need to discuss all participants monthly, and alerted supervisors to participants not responding to treatment.

#### Usual care

Participants received care from their general practitioner according to usual clinical practice for these patients, including treatment by antidepressants and referral for other treatments. We recorded every aspect of usual care but did not specify a treatment programme in line with the pragmatic nature of this trial.

### Outcomes

Our primary outcome was individual participant depression severity measured by the patient health questionnaire 9 (PHQ-9)^22^ at four months. Secondary outcomes were the PHQ-9 at 12 months; quality of life (short form 36 questionnaire; SF-36) at baseline, four months, and 12 months^23^; worry and anxiety (generalised anxiety disorder; GAD-7)^24^ at four and 12 months; and patient satisfaction (client satisfaction questionnaire 8; CSQ-8)^25^ at four months.

### Sample size

We powered the trial at 90% (α=0.05) to detect an effect size of 0.4, which we regarded as reasonable for determining clinically meaningful differences between interventions.^26^ This figure was within the 95% confidence intervals of the effect predicted from data collected during our pilot work (effect size 0.63; 95% confidence interval 0.18 to 1.07).^13^ However, the figure was greater than the findings from a meta-analysis of existing trials available at the time we initiated CADET (0.25; 0.18 to 0.32).^7^ We would have required 132 participants per group in a two armed, patient randomised trial. For our cluster trial, with 12 patients per primary care cluster and an intracluster correlation of 0.06 from our pilot trial,^13^ the design effect was 1.65, leading to a sample size of 440. To follow up 440 participants, we aimed to randomise 550 participants (anticipating 20% attrition). Because recruitment would not be uniform between practices, we aimed to recruit 48 practices with up to 14 participants per practice.

### Statistical methods

We undertook intention to treat analyses for all outcomes, reported in accordance with CONSORT guidelines.^27^ All analyses were undertaken in Stata 10.1, after a predefined analysis plan agreed with the trial steering committee. We analysed available data at four and 12 months by ordinary least squares or logistic regression. This approach allowed for clustering by use of robust standard errors, adjusted at the cluster level for minimisation variables and site; at the individual level for age; and, where appropriate, the baseline measurement of the variable. We analysed the effect of missing primary and secondary outcome data as a sensitivity analysis, estimated by multiple imputation by chained regression equations^28^ using all available scale clinical scores, age, sex, practice variables, site, and treatment group. To ease interpretation and allow comparison with published studies, we estimated standard effect sizes using the baseline standard deviation for all participants and calculated rates of “recovery” (proportions of participants with PHQ-9 scores ≤9) and “response” (50% reduction in scores from baseline). We calculated numbers needed to treat from the inverse of the absolute risk reduction adjusted for clustering by practice.

## Results

### Participant flow and retention

We allocated 53 practices, two of which dropped out after allocation (fig[Fig fig1]). These practices were removed from the minimisation schedule and their data did not influence later allocations. During recruitment, we found that the cut-off adopted for the Index of Multiple Deprivation had been set far too high—with all practices so far recruited being below it. We changed this cut-off to one close to the median of practices so far recruited, retaining allocations so far. One practice was found to have been mistakenly recorded in the wrong geographical area; it was moved to the correct group, retaining its allocation. Table 1 shows the final balance achieved. Of the remaining 51 practices, two did not recruit any participants. The mean number of participants recruited for the remaining 49 practice clusters was 11.9 (standard deviation 3.9, range 4-20). We recruited 581 participants in total and followed up 505 (87%) and 498 (86%) at four and 12 months, respectively.

**Figure fig1:**
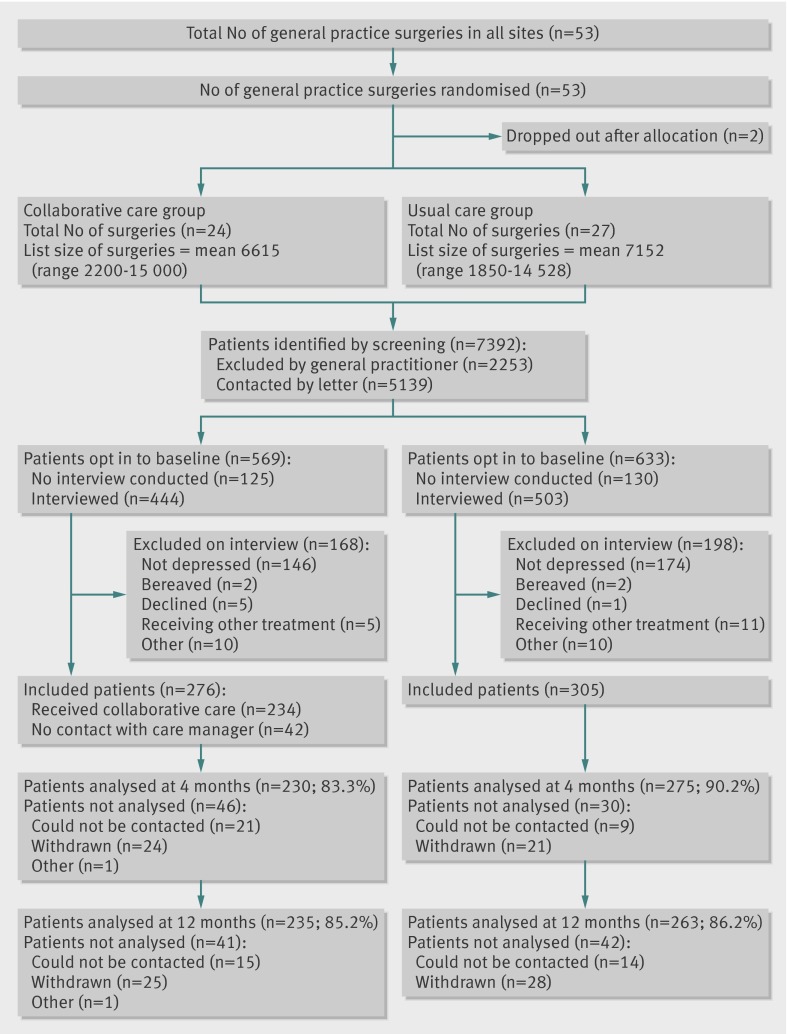
CONSORT diagram. *The five patients in the collaborative care group who were excluded on interview because they were receiving treatment from secondary care or another mental health provider (n=5) included one participant who was initially allocated in error and subsequently excluded

**Table 1 tbl1:** Baseline characteristics of participants

	Collaborative care (n=276)	Usual care (n=305)	Total (n=581)
No of general practices by centre			
Bristol	8	9	17
London	9	9	18
Manchester	7	9	16
Minimisation variables			
Mean Index of Multiple Deprivation (SD)	9210 (7416)	8449 (6012)	8807 (6651)
Mean no of general practitioners (SD)	3.8 (2.0)	4.0 (1.9)	3.9 (1.9)
Mean no of patients (SD)	6615 (3282)	7152 (3781)	6899 (3530)
Sex			
Female	202 (73.2)	216 (70.8)	418 (71.9)
Male	74 (26.8)	89 (29.2)	163 (28.1)
Age (years)			
Mean (SD)	45.0 (13.2)	44.5 (13.4)	44.8 (13.3)
Range	18-82	17-79	17-82
Ethnic origin			
White British	233 (84.4)	261 (85.6)	494 (85.0)
Other	43 (15.6)	44 (14.4)	87 (15.0)
Education			
None	54 (19.6)	74 (24.3)	128 (22.0)
GCSE or O level	65 (23.6)	81 (26.6)	146 (25.1)
Post-GCSE or O level	84 (30.4)	79 (25.9)	163 (28.1)
Degree or higher	49 (17.8)	53 (17.4)	102 (17.6)
Other or don’t know	24 (8.7)	18 (5.9)	42 (7.2)
Employment			
Employed or self employed	130 (47.4)*	122 (40.0)	252 (43.5)*
Not working	144 (52.6)	183 (60.0)	327 (56.5)
Marital status			
Married or cohabiting	127 (46.0)	114 (37.4)	241 (41.5)
Antidepressant treatment			
Prescribed antidepressants	231 (83.7)	249 (81.6)	480 (82.6)
Revised Clinical Interview Schedule score			
Mean (SD)	28.8 (9.3)	30.3 (8.9)	29.6 (9.1)
ICD-10 diagnosis†			
Mild	42 (15.2)	41 (13.4)	83 (14.3)
Moderate	156 (56.5)	167 (54.8)	323 (55.6)
Severe	78 (28.3)	96 (31.5)	174 (29.9)
History of depression			
Previous episode	202 (73.2)	220 (72.1)	422 (72.6)
Secondary diagnosis			
Any anxiety disorder	269 (97.5)	301 (98.7)	570 (98.1)
Longstanding physical illness	171 (62.0)	199 (65.2)	370 (63.7)

Data are number (%) of participants unless stated otherwise. SD=standard deviation.

*Employment data were missing for two participants.

†One participant did not meet ICD-10 criteria for mild, moderate, or severe depression on the revised Clinical Interview Schedule score.

### Baseline characteristics of participants

More than half (56%) of participants fulfilled ICD-10 criteria for a moderately severe depressive episode, with a further 30% meeting criteria for severe depression, 14% mild depression, and 73% having had depression in the past (table 1). Fewer than half (44%) of participants were in full or part time paid employment, the mean age was 44.8 years (standard deviation 13.3), and 72% were women. Almost all (98%) participants had a secondary diagnosis of an anxiety disorder, the most common being generalised anxiety disorder. Almost two thirds of participants (64%) reported a longstanding physical illness (for example, diabetes, asthma, heart disease). At baseline, 83% of participants had been prescribed antidepressant drugs by their primary care doctor.

### Delivery and receipt of the intervention

Ten care managers provided collaborative care for a mean of 27.6 participants each (standard deviation 16.42, range 4-46). Patients received a mean number of 5.6 sessions (4.01, 0-15). Forty two (15.2%) participants did not attend any sessions with their care manager, 213 (77.2%) had two or more contacts, and 171 (62.2%) had four or more contacts. The mean total time in collaborative care was 3.03 hours (standard deviation 2.18) over a period of 12 weeks (7.75). For those participants who attended at least one session, the mean duration of sessions was 34.5 minutes (8.2). Most participants in both collaborative care and usual care groups remained on antidepressant prescriptions (74.8% *v* 73.8% at four months; 69.7% *v* 69.2% at 12 months).

### Primary outcome

The mean depression score at four months was 1.33 PHQ-9 points lower (95% confidence interval 0.35 to 2.31, P=0.009) in participants receiving collaborative care than those receiving usual care (table 2) after adjustment for baseline depression. This difference equated to a standardised effect size of 0.26 (0.07 to 0.46). More participants receiving collaborative care than those receiving usual care met criteria for recovery (odds ratio 1.67 (95% confidence interval 1.22 to 2.29); number needed to treat=8.4) and response (1.77 (1.22 to 2.58); 7.8; table 3).

**Table 2 tbl2:** Intention to treat analysis of primary and secondary outcomes at four and 12 month follow-up

	Collaborative care			Usual care		Adjusted difference (95% CI)	P	Effect size
	No of participants	Mean (SD)		No of participants	Mean (SD)			
Primary outcome								
PHQ-9, baseline	276	17.4 (5.2)		305	18.1 (5.0)	—	—	—
PHQ-9, 4 months*	230	11.1 (7.3)		275	12.7 (6.8)	−1.33 (−2.31 to −0.35)	0.009	0.26
Secondary outcomes								
PHQ-9, 12 months*	235	10.0 (7.1)		263	11.7 (6.8)	−1.36 (−2.64 to −0.07)	0.04	0.28
GAD-7, baseline	276	12.9 (5.3)		305	13.6 (4.7)	—	—	—
GAD-7, 4 months	228	9.1 (6.8)		273	9.8 (5.8)	−0.39 (−1.30 to 0.53)	0.4	0.08
GAD-7, 12 months	227	7.7 (6.2)		253	9.1 (6.2)	−1.09 (−2.21 to 0.03)	0.06	0.22
SF-36 MCS, baseline	276	23.2 (10.4)		305	22.3 (10.3)	—	—	—
SF-36 MCS, 4 months	227	34.6 (15.4)		268	30.7 (13.7)	3.4 (1.1 to 5.7)	0.005	0.33
SF-36 MCS, 12 months	223	36.4 (15.0)		249	33.4 (14.5)	2.5 (−0.6 to 5.5)	0.1	0.24
SF-36 PCS, baseline	276	44.8 (12.4)		305	44.5 (12.3)	—	—	—
SF-36 PCS, 4 months	227	45.8 (13.2)		268	45.6 (13.8)	0.05 (−1.67 to 1.56)	0.9	-0.004
SF-36 PCS, 12 months	223	46.1 (13.2)		249	44.9 (13.3)	1.04 (−0.93 to 3.01)	0.3	0.08
CSQ-8, 4 months	232	25.3 (5.8)		269	22.1 (6.2)	3.13 (1.87 to 4.39)	<0.001	0.52

SD=standard deviation; MCS=mental component; PCS=physical component.

*One participant committed suicide. Because the PHQ-9 data for this person could not be regarded as missing at random, PHQ-9 data at four and 12 months were set to the maximum 27 and included in analysis.

**Table 3 tbl3:** Recovery, response, and numbers needed to treat*

	Collaborative care			Usual care		Odds ratio† (95% CI‡)	P‡	No needed to treat§
	No of participants	No (%) of recovered or responded participants		No of participants	No (%) of recovered or responded participants			
Recovery (PHQ-9 score ≤9 at follow-up)								
4 months	230	108 (47.0)		275	96 (34.9)	1.67 (1.22 to 2.29)	0.001	8.4
12 months	235	131 (55.7)		263	106 (40.3)	1.88 (1.28 to 2.75)	0.001	6.5
Response (≥50% reduction in PHQ-9 score at follow-up *v* PHQ-9 score at baseline)								
4 months	230	99 (43.0)		275	83 (30.2)	1.77 (1.22 to 2.58)	0.003	7.8
12 months	235	115 (48.9)		263	93 (35.4)	1.73 (1.22 to 2.44)	0.002	7.3

*One participant committed suicide. Because the PHQ-9 data for this person could not be regarded as missing at random, PHQ-9 data at four and 12 months were set to the maximum 27 and included in analysis.

†Adjusted for age, site, and minimisation variables.

‡Adjusted for clustering by practice.

§For numbers needed to treat, inverse of absolute risk reduction adjusted for clustering by practice.

### Secondary outcomes

At 12 month follow-up, mean PHQ-9 score was 1.36 points lower (95% confidence interval 0.07 to 2.64, P=0.04) in participants receiving collaborative care than in those receiving usual care (standardised effect size 0.28 (95% confidence interval 0.01 to 0.52)). More participants in collaborative care than those in usual care met criteria for recovery (odds ratio 1.88 (95% confidence interval 1.28 to 2.75); number needed to treat=6.5) and response (1.73 (1.22 to 2.44); 7.3; tables 2 and 3).

Collaborative care produced better outcomes than usual care on the mental component scale of the SF-36 at four but not 12 months, had little effect on anxiety and the physical component scale of the SF-36, and participants receiving collaborative care were more satisfied with their treatment than those in usual care (table 2).

### Missing data

Multiple imputation data using least squares regression and 100 multiple datasets for the primary outcome produced an imputed estimate that was similar to the available data (PHQ-9 coefficient −1.31 (95% confidence interval −2.37 to −0.26), P=0.02). We also found very little difference between the results of our analyses of available data and imputed data on any secondary outcomes at four and 12 month follow-up. This similarity showed that all analyses were not substantively affected by missing data or differential rates of follow-up between trial arms.

## Discussion

We found that collaborative care improves depression immediately after treatment compared to usual care, which has effects that persist to 12 month follow-up and is preferred by patients over usual care. Our observed effect size (0.26) was less than that used to power the study, although the 95% confidence intervals around it (0.07 to 0.46) encompassed our original target effect size (0.4). Our result was also within the 95% confidence interval of the standardised mean difference found in the 2012 Cochrane meta-analysis of 79 randomised controlled trials (overall standardised mean difference 0.29, 95% confidence interval 0.23 to 0.36).^6^ This analysis included our results, and was no different from trials in the US (0.28, 0.21 to 0.35), non-US regions excluding the UK (0.36, 0.13 to 0.59), and the UK (0.32, 0.07 to 0.57). Collaborative care is as effective in the UK healthcare system—an example of an integrated health system with a well developed primary care sector—as in the US. Our study adds to the emerging international literature from countries such as Chile^9^ and India^10^ indicating that collaborative care is a model that reliably generalises outside the US.

### Strengths and limitations of the study

To our knowledge, CADET is the one of the largest studies of collaborative care. Less than 50% of published collaborative care trials have followed up participants for 12 or more months, and our levels of attrition at four and 12 months are comparable with 70% of collaborative care trials and better than other trials of brief interventions in this area.^29^ There was no evidence that missing follow-up data biased findings. Although our cluster design protected against contamination of the usual care arm by changes in behaviour being tested in the collaborative care arm, cluster trials are prone to selection bias. We minimised this bias by recruiting participants through electronic case note searches rather than doctor referral.

Owing to the nature of the intervention and comparator, we could not blind general practitioners, patients, or care managers to treatment allocation. However, we used self reported outcome measures to minimise the effect of detection bias. We relied on self reported records of care manager contacts, and thus have no means to assess record accuracy. Care managers were already employed by organisations providing primary care services for mental health in the UK’s health service. However, supervisors were senior members of the investigator group, so it is unclear how much their—albeit minimal—supervision can be generalised beyond the trial. 

Our intervention was brief, so a more intensive intervention might have improved outcomes further, particularly for the more complex cases. We could have chosen a different psychological intervention such as cognitive behavioural therapy,^30^ but reviews^19^ and controlled trials^31^ have shown behavioural activation to be as effective as cognitive behavioural therapy—potentially more so for severe cases^32^—and can be delivered effectively by junior healthcare personnel.^31^ We are currently conducting a process evaluation including analysis of any dose response, to determine the effect on outcomes of intervention content, process mediators, and participant characteristics.

### Implications for practice and directions for future research

During the time we undertook CADET, the number of international trials of collaborative care more than doubled, albeit many of them still conducted in the US. While the portability of a US collaborative care model to the UK had been suggested by previous small scale studies, CADET has provided definitive confirmation of that portability. The CADET trial findings answered a specific need highlighted in the NICE guidelines for depression and provided critical evidence for further service delivery.

Although our results sit within the expected effect range of collaborative care reported in the latest meta-analysis of international collaborative care trials,^6^ the clinical implications of our results are more difficult to interpret. The average difference in treatment response was less than what we had expected (effect size 0.26 *v* 0.4), and these more modest differences were sustained over the longer term. Between group differences can obscure response rates in individual patients. We have, therefore, presented the data on meaningful clinical differences (table 3) using numbers needed to treat and two criteria commonly applied in the depression literature and regarded as clinically meaningful. These criteria were recovery (falling below a recognised point on the PHQ-9 symptom scale); and response (a 50% reduction in symptoms of depression). 

Using these metrics, it is particularly noteworthy that at 12 months, 56% of participants receiving collaborative care “recovered”—15% more than in usual care. Health services would, therefore, need to treat 6.5 patients using collaborative care to produce one additional patient with a sustained recovery compared with usual care. Studies achieving higher effects have been undertaken in countries with less developed services in primary care than in the UK,^9^ ^10^ or have used more highly qualified workers such as nurses or social workers.^33^ Such workers are in acute short supply in the UK and hence this model would not have been translatable for its health system. A full economic evaluation of CADET is forthcoming, since the relative magnitude of clinical effects can only be properly understood in the context of a proper understanding of cost data relative to effectiveness. The persistence of our treatment effect at 12 months, however, is noteworthy and unusual in primary care depression trials.^29^ We are now conducting a three year follow-up of our participants to assess further long term effects, given that only two trials of collaborative care have reported outcomes beyond 24 months.^6^

Our results represent a slightly higher recovery rate than that reported by the UK Improving Access to Psychological Therapies (IAPT) programme, which has been described in a *Nature* editorial as “a world-beating standard.”^34^ Recovery rates of about 45% have been achieved in IAPT, after an investment of £700m (€812m; $1085m) over six years.^35^ We suggest that integration of our CADET protocol into IAPT services might enhance outcomes for patients receiving treatment for depression and provide guidance to international mental health services that this model can be applicable outside the US.

Although collaborative care is an organisational intervention that improves outcomes, much remains to be done to improve the effectiveness of treatments for depression. Even intensive psychological treatments for depression have been shown to achieve only modest gains (effect size of 0.42 in 51 studies).^36^ Our careful selection of intervention ingredients, directed by our identification of components present in the better performing trials from our previous meta-regression,^15^ did not succeed in achieving the larger effects we had hoped for. In CADET, 44% of participants receiving collaborative care had scores that remained above the PHQ-9 depression threshold at 12 month follow-up. Future trials should test enhancements of the basic collaborative care model by developing, testing, and delivering better treatments within the effective collaborative care organisational framework, rather than test collaborative care itself, given that the effects of collaborative care are now firmly established.

**What is already known on this topic**

Although systematic reviews have shown that collaborative care improves depression outcomes, most studies have been conducted in the US
In other areas of mental healthcare, the positive effects of organisational interventions developed in the US have not generalised outside the original healthcare context
Despite some preliminary evidence in non-US regions, there is uncertainty in meta-analyses around the true effect of collaborative care outside the US

**What this study adds**

Collaborative care improves depression immediately after treatment compared with usual care, which has effects persisting up to 12 month follow-up and is preferred by patients over usual care
The portability of a model of collaborative care in the US had been suggested by small scale studies; the CADET trial has provided a definitive confirmation of that portability
Although collaborative care is an organisational intervention that improves outcomes, better treatments are still needed to increase recovery rates in depression above the current 50-55%

## Notes

Contributors: DAR, LG, KL, CC-G, PB, JC, SP, RA, DK, JMB, CG, SG, GL, CM, and MB designed the study and were responsible for its conduct. JJH and AH-M were responsible for study management and data collection; JMB, CG, DAR, and JJH undertook data analysis. All authors had full access to all of the data (including statistical reports and tables) in the study and can take responsibility for the integrity of the data and the accuracy of the data analysis. All authors contributed to the writing and editing of the manuscript. DAR is guarantor.

Ethical approval: Ethical approval for the study was given by the NHS Health Research Authority, NRES Committee South West (NRES/07/H1208/60). All participants gave informed consent before taking part in the study.

Data sharing: patient level data is available from the corresponding author. Consent for data sharing was not obtained but the presented data are anonymised and risk of identification is low.

The lead author affirms that this manuscript is an honest, accurate, and transparent account of the study being reported; that no important aspects of the study have been omitted; and that any discrepancies from the study as planned (and, if relevant, registered) have been explained.

## Notes

Cite this as: *BMJ* 2013;347:f4913
